# Fatty Acids Pattern, Essential Oil Content and Constituents of Some Sage Species (*Salvia* sp.) Native to Iran: Usable in Food Industry

**DOI:** 10.1002/fsn3.71308

**Published:** 2025-11-30

**Authors:** Yasaman Veis Mohammadi, Jalal Khorshidi, Farzad Nazari

**Affiliations:** ^1^ Department of Horticultural Science and Engineering Faculty of Agriculture, University of Kurdistan Sanandaj Iran

**Keywords:** germacrene D, linoleic acid, linolenic acid, *Salvia*

## Abstract

The genus *Salvia* is a rich source of bioactive compounds with diverse applications in traditional medicine, as well as in the food, and pharmaceutical industries. This study evaluated the essential oil content and composition, seed oil yield, and fatty acid profiles of five native Iranian *Salvia* species ( *S. nemorosa* , *S. spinosa* , *S. syriaca* , *S. palaestina* , and *S. multicaulis* ) cultivated under uniform ecological conditions. Essential oils were extracted using a Clevenger apparatus based on the hydrodistillation method, while seed oils were obtained via Soxhlet extraction with hexane as the solvent. Both were subsequently analyzed by GC–MS and GC‐FID. Results revealed significant interspecific variation. Essential oil content ranged from 0.014% (v/w) in *S. nemorosa* to 0.37% (v/w) in *S. multicaulis* . In contrast, the highest seed oil content (53.3% v/w) was found in *S. nemorosa* , while the lowest (18% v/w) was recorded in *S. syriaca* . *S. nemorosa* was rich in spathulenol and caryophyllene derivatives, whereas *S. spinosa* was dominated by isopentyl isovalerate. *S. syriaca* and *S. palaestina* primarily contained germacrene D, while *S. multicaulis* showed high levels of isoborneol, bornyl acetate, and 1,8‐cineole. Palmitic, stearic, linoleic, and linolenic acids were detected in the seed oil of all studied *Salvia* species. *S. multicaulis* exhibited the highest levels of palmitic (10.92% ± 0.33% v/w) and linoleic acid (52.57% ± 4.57% v/w), *S. palaestina* had the highest stearic acid content (2.92% ± 0.045% v/w), and *S. nemorosa* contained the highest linolenic acid level (46.3% ± 0.594% v/w). Overall, *S. multicaulis* stood out for its high essential oil content, rich in isoborneol and bornyl acetate, whereas *S. nemorosa* exhibited the highest seed oil content dominated by linolenic acid. These findings suggest that *S. multicaulis* and *S. nemorosa* are promising candidates for essential oil and edible oil production, respectively, offering valuable potential for application in the pharmaceutical, cosmetic, and food industries.

## Graphical abstract

This study investigated the essential oil and seed oil characteristics of five native Iranian Salvia species cultivated under uniform conditions. Significant interspecific variation was observed in oil content and composition, with *S. multicaulis* and *S. nemorosa* identified as promising sources for essential oil and fixed oil production, respectively. These findings highlight their potential applications in pharmaceutical, food, and cosmetic industries.

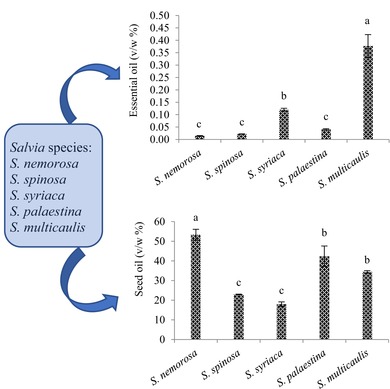

## 1 Introduction

Sage (*Salvia* sp.) is the largest genus in the Lamiaceae family, comprising approximately 1000 species distributed across various regions of the world (Ortiz‐Mendoza et al. 2022). In Iran, this genus includes about 60 species, 17 of which are endemic (Askari et al. 2021). Various *Salvia* species have long been used as flavoring agents and for the treatment of different diseases in traditional medicine (Ghorbani and Esmaeilizadeh 2017). Studies have demonstrated that sage (in powder or extract form) helps preserve the quality of meat products during storage (Mizi et al. 2019; Cegiełka et al. 2022). In Iranian traditional medicine, sage is used as an antiseptic, diuretic, stomach tonic, and anti‐flatulent, and for treating diarrhea, indigestion, fever, rheumatism, cough, and the common cold (Naghibi et al. 2005). Furthermore, previous studies have confirmed the antioxidant, anti‐inflammatory (Guy et al. 2010), antimicrobial (Shanaida et al. 2021), antidiabetic (Eidi and Eidi 2009), anticancer (Semiz et al. 2023), antiviral (Abou Baker et al. 2021), and antidepressant (Seol et al. 2010) activities of various *Salvia* species.

Terpenoids (such as α‐thujone, camphor, 1,8‐cineole, α‐humulene, β‐caryophyllene, and viridiflorol), anthocyanins, coumarins, flavonoids (including luteolin, apigenin, kaempferol, and quercetin), and phenolic acids (such as caffeic acid, rosmarinic acid, and salvianolic acid), represent the most valuable secondary metabolites in the *Salvia* genus (Demirpolat 2023; Lopresti 2017). Additionally, the seeds of *Salvia* species contain varying amounts of fixed oil rich in fatty acids such as linolenic, linoleic, oleic, palmitic, and stearic acids (Moazzami Farida et al. 2016).

Numerous studies have investigated the quantity and quality of essential oils and fixed oils in various *Salvia* species. However, most of these studies focused on plant samples collected from natural habitats with differing ecological conditions (Nejad Habibvash et al. 2007; Kurşat et al. 2013; Moazzami Farida et al. 2016; Hatipoglu et al. 2016; Demirpolat 2023; Jazayeri Gharehbagh et al. 2023). Since the production and accumulation of primary and secondary metabolites in plants are influenced by both genetic and environmental factors (Khorshidi et al. 2020), identifying superior species based on target compounds is only possible when all species are cultivated under similar ecological conditions. Therefore, in the present study, the essential oil content and composition of the aerial parts, as well as the fixed oil content and fatty acid profiles of seeds from several native Iranian *Salvia* species, were compared under the same cultivation conditions. The objective was to identify the superior species in terms of target phytochemical constituents and to introduce them as potential candidates for use in the food and pharmaceutical industries.

## 2 Materials and Methods

### 2.1 Seed Collection and Sowing of the Studied *Salvia* Species

Seeds of *S. nemorosa* , *S. spinosa* , *S. syriaca* , *S. palaestina* , and *S. multicaulis* species were collected at full maturity stage from their natural habitats in Sanandaj, Kurdistan Province, Iran, in June 2022. The voucher specimens, numbered 1864, 1928, 3044, 6920, and 2083 for *S. nemorosa* , *S. spinosa* , *S. syriaca* , *S. palaestina* , and *S. multicaulis* , respectively, were deposited in the HKS Herbarium of the Kurdistan Agricultural and Natural Resources Research and Education Center, Sanandaj, Iran. After shade‐drying, seeds were stored at room temperature under ambient humidity conditions until sowing. Then, the seeds were sown in the field during autumn in a randomized complete block design (RCBD) with three replications. Each experimental plot was 4 m^2^ (2m × 2 m), with a row spacing of 40 cm and a plant spacing of 20 cm within rows. Irrigation, thinning, and weed control were performed uniformly and regularly for all species throughout the study period.

### 2.2 Plant Harvesting and Essential Oil Extraction

Plant samples of each *Salvia* species were harvested at full flowering stage and shade‐dried. Essential oils were extracted from the dried aerial parts using a Clevenger‐type apparatus based on the hydrodistillation method. Distillation was carried out for 3 h with three replications per species. After distillation, the essential oil volume was measured, and its content (v/w %) was calculated. Then, the obtained essential oils were dehydrated with anhydrous sodium sulfate, poured into glass vials, and stored at 4°C until analysis (Rustaiyan et al. 2005).

### 2.3 Seed Harvesting and Oil Extraction

At the fruit ripening stage, seeds were harvested, shade‐dried and powdered by grinder. Oil extraction from powdered seeds was performed using a Soxhlet apparatus with *n*‐hexane as the solvent for 3 h. The obtained mixture was filtered, and the solvent was completely evaporated using a rotary evaporator (50°C, 15 min) and oven (40°C for 48 h) (Moazzami Farida et al. 2016). Fatty acid methyl esterification was carried out using methanolic KOH (2 M) at 40°C for 20 min. Then, the obtained fatty acid methyl esters were extracted with hexane, and the samples were stored at 4°C until analysis (Bagci et al. 2004).

### 2.4 Essential Oil and Oil Analysis

Essential oils and seed oils were analyzed using a gas chromatography–mass spectrometry (GC/MS) and gas chromatography equipped with a flame ionization detector (GC/FID). The specifications and operating conditions for both GC/MS and GC/FID were as follows (Khorshidi et al. 2024):

GC/MS: An Agilent gas chromatograph equipped with an HP‐5MS column (30 m length, 0.25 mm internal diameter, 0.25 μm film thickness) was used. The column's initial temperature was set at 50°C and held for 2 min at this temperature, then raised to 260°C at a rate of 7°C/min, and held at 260°C for 8 min. The ionization energy was 70 eV, and the mass scanning range was 50–550 amu. The injection chamber and ionization source temperatures were set at 280°C and 230°C, respectively. Helium (99.99% purity) was used as the carrier gas at a pressure of 34 psi and a flow rate of 1 mL/min.

GC/FID: An Agilent gas chromatograph series 7890‐B equipped with a flame ionization detector (FID) was used. The detector temperature was set at 290°C. The injection chamber temperature, column specifications, temperature program, and carrier gas characteristics were the same as those used in the GC/MS analysis.

To identify the essential oils and oil components, the obtained mass spectra were compared with data from the NIST and Wiley libraries. The quantification of the components was performed using relative area percentages obtained from the GC‐FID analysis.

### 2.5 Statistical Analysis

All data were analyzed using SPSS software (version 25). Means were compared using Duncan's multiple range test at a significance level of *p* ≤ 0.05.

## 3 Results

### 3.1 Essential Oil and Oil Content

**FIGURE 1:**
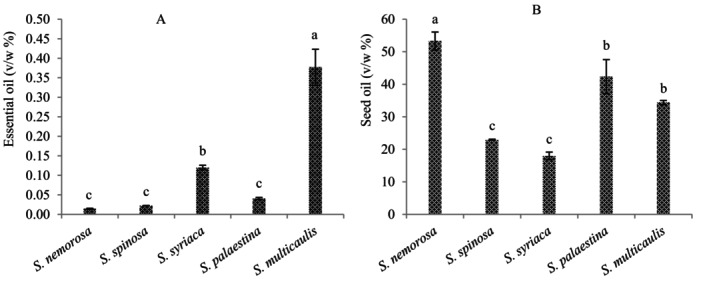
Essential oil content (A) and seed oil content (B) of the studied *Salvia* species. Columns with the same letters are not significantly different at the 5% probability level based on the Duncan’s multiple range test.

The studied *Salvia* species exhibited significant variations in their essential oil content. The highest essential oil content (0.37% v/w) was obtained from *S. multicaulis* , followed by *S. syriaca* (0.12% v/w). No significant differences were detected in the essential oil content of *S. palaestina* (0.04% v/w), *S. spinosa* (0.022% v/w), and *S. nemorosa* (0.014% v/w) (Figure 1A). Similarly, the seed oil content showed notable differences among the *Salvia* species. The highest seed oil content (53.3% v/w) was recorded in *S. nemorosa* , followed by *S. palaestina* (42.35% v/w) and *S. multicaulis* (34.4% v/w). In contrast, the lowest seed oil content (18% v/w) was found in *S. syriaca* , which did not differ significantly from *S. spinosa* (22.95% v/w) (Figure 1B).

### 3.2 Essential Oil Composition

The number, type, and dominant constituents of the essential oils varied considerably among the studied *Salvia* species. A total of 23, 68, 34, 63, and 60 compounds were identified in the essential oils of *S. nemorosa* , *S. spinosa* , *S. syriaca* , *S. palaestina* , and *S. multicaulis* , respectively. The dominant constituents of essential oils in the studied *Salvia* species were as follows:

*S. nemorosa* : (+)‐spathulenol (23.21 ± 0.27), trans‐caryophyllene (21.92 ± 0.19), caryophyllene oxide (17.18 ± 0.2), and bicyclogermacrene (7.75 ± 0.08) (Table 1). *S. spinosa* : isopentyl isovalerate (15.02 ± 0.787), β‐eudesmol (9.66 ± 0.388), trans‐caryophyllene (6.85 ± 0.323), 2‐methylbutyl isovalerate (5.81 ± 0.282), and β‐ocimene (5.05 ± 0.266) (Table 2). *S. syriaca* : germacrene D (16.17 ± 0.183), bicyclogermacrene (10.58 ± 0.072), and (+)‐spathulenol (8.47 ± 0.058) (Table 3). *S. palaestina* : germacrene D (21.45 ± 0.55), caryophyllene oxide (12.53 ± 2.56), trans‐caryophyllene (11.97 ± 0.364), and linalool (10.14 ± 0.34) (Table 4). *S. multicaulis* : isoborneol (11.86 ± 0.02), bornyl acetate (10.96 ± 0.01), 1,8‐cineole (10.48 ± 0.07), trans‐caryophyllene (9.74 ± 0.08), camphor (9.18 ± 0.049), and α‐pinene (8.67 ± 0.07) (Table 5).

**TABLE 1:** Essential oil composition of *Salvia nemorosa* species.

Component	Retention time (min)	%
Sabinene	5.58	1.77 ± 0.02
Borneol	9.65	2.39 ± 0.02
Terpinen‐4‐ol	9.90	0.341 ± 0.34
Bornyl acetate	12.05	2.46 ± 0.03
Sabinyl acetate	12.18	0.78 ± 0.01
Bicycloelemene	13.05	2.58 ± 0.03
trans‐Caryophyllene	14.65	21.92 ± 0.19
β‐Selinene	15.26	2.08 ± 0.01
Germacrene D	15.75	0.9 ± 0.01
β‐Ionone	15.82	0.41 ± 0.4
Cycloheptasiloxane, tetradecamethyl—	15.89	2.57 ± 0.43
Bicyclogermacrene	16.03	7.75 ± 0.08
(+)‐Spathulenol	17.49	23.21 ± 0.27
Caryophyllene oxide	17.56	17.18 ± 0.2
3‐Cyclohexen‐1‐carboxaldehyde, 3,4‐dimethyl—	17.99	0.83 ± 0.01
(−)‐Spathulenol	18.45	2.95 ± 0.05
Aromadendrene	18.57	0.41 ± 0.4
Tetracosamethyl‐cyclododecasiloxane	18.76	2.47 ± 0.2
α‐Costol	19.01	2.06 ± 0.18
Octadeamethyl‐cyclononasiloxane	21.22	0.411 ± 0.41
2‐Pentadecanone	21.50	1.13 ± 0.08
1,2‐Benzenedicarboxylic acid	21.91	0.251 ± 0.25
Phytol	25.22	3.1 ± 0.01

**TABLE 2:** Essential oil composition of *Salvia spinosa* species.

Component	Retention time (min)	%
Propanoic acid, 2‐methyl‐, 2‐methylpropyl ester	4.39	0.21 ± 0.002
α‐Pinene	4.79	0.47 ± 0.02
β‐Phellandrene	5.55	0.23 ± 0.048
1‐Octen‐3‐ol	5.69	0.092 ± 0.091
β‐Pinene	5.89	1.97 ± 0.05
Isobutyl 2‐methylbutyrate	6.12	0.15 ± 0.002
Isobutyl isovalerate	6.18	0.64 ± 0.023
Isoamyl isobutanoate	6.32	3.91 ± 0.198
2‐Methylbutyl isobutyrate	6.39	2 ± 0.089
dl‐Limonene	6.68	0.29 ± 0.023
trans‐β‐Ocimene	6.85	1.06 ± 0.057
β‐Ocimene	7.09	5.05 ± 0.266
Isopentyl 2‐methylbutanoate	8.18	4.69 ± 0.248
Isopentyl isovalerate	8.33	15.02 ± 0.787
2‐Methylbutyl isovalerate	8.38	5.81 ± 0.282
Allo ocimene	8.80	0.071 ± 0.07
Pentyl isovalerate	9.08	0.43 ± 0.014
n‐Hexyl isobutyrate	9.18	0.83 ± 0.039
Isoamyl angelate	10.16	0.73 ± 0.042
Myrtenal	10.25	0.081 ± 0.08
2‐Methylpentanol‐1	10.38	0.7 ± 0.041
2,6‐Dimethyl‐3,5,7‐octatriene‐2‐ol, E, E—	10.54	0.43 ± 0.025
β‐Cyclocitral	10.74	0.17 ± 0.007
cis‐3‐Hexenyl‐α‐methylbutyrate	10.91	0.15 ± 0.008
Butyric acid, 2‐methyl‐, hexyl ester	11.00	1.83 ± 0.094
n‐Hexyl iso‐valerate	11.10	3.49 ± 0.183
Isopentyl hexanoate	11.26	0.23 ± 0.008
Isobutyrone	11.39	2.46 ± 0.115
Bicycloelemene	13.02	0.32 ± 0.048
Hexylene glycol	13.13	0.39 ± 0.023
α‐Copaene	13.79	0.99 ± 0.098
β‐Damascenone	13.94	0.91 ± 0.049
Benzyl isovalerate	14.13	0.92 ± 0.025
α‐Barbatene	14.46	0.29 ± 0.012
Dihydrocurcumene	14.53	0.49 ± 0.011
trans‐Caryophyllene	14.64	6.85 ± 0.323
n‐Butyl 2‐methylbenzoate	14.92	3.7 ± 0.137
β‐Barbatene	15.08	0.57 ± 0.004
Neryl acetone	15.15	0.55 ± 0.0
β‐Selinene	15.25	0.52 ± 0.002
Amorpha‐4,11‐diene	15.33	0.082 ± 0.081
Germacrene D	15.74	2.06 ± 0.09
β‐Ionone	15.79	1.61 ± 0.07
Phensuximide	15.89	1.47 ± 0.053
Germacrene B	16.00	0.46 ± 0.004
α‐Farnesene	16.12	0.35 ± 0.0
Cuparene	16.17	0.191 ± 0.19
Dihydro‐β‐agarofuran	16.42	1.35 ± 0.001
(1S*,6R*,7S)‐7‐Tricyclo[5.3.2.0 (1,6)]dodecan‐7‐ol	16.52	1.11 ± 0.178
Elemol	16.92	0.17 ± 0.001
Isoaromadendrene epoxide	17.00	0.091 ± 0.09
Nerolidol	17.10	0.074 ± 0.07
Caryophyllene oxide	17.53	3.11 ± 0.129
Hexadecane	17.62	0.451 ± 0.45
α‐Eudesmol	17.73	0.24 ± 0.008
2‐Methylbutyl‐3‐phenyl‐propionate	17.80	0.59 ± 0.027
Benzenepropanoic acid, pentyl ester	17.86	0.49 ± 0.022
γ‐Eudesmol	18.15	0.23 ± 0.008
Isospathulenol	18.30	0.83 ± 0.067
Isoaromadendrene epoxide	18.43	0.11 ± 0.104
Valencene	18.54	1.75 ± 0.055
β‐Eudesmol	18.69	9.66 ± 0.388
Trichloro (2‐pentenyl) silane	18.93	0.191 ± 0.19
Spiro[4.5]decan‐7‐one, 1,8‐dimethyl‐8,9‐epoxy‐4‐isopropyl—	19.42	0.081 ± 0.08
Hexahydro farnesyl acetone	21.48	0.851 ± 0.85
Phthalic acid	21.88	0.221 ± 0.22
Tricyclo [4.3.0.0 (7,9)]nonane, 2,2,5,5,8,8‐hexamethyl‐, (1α., 6β., 7α., 9.α.)—	22.57	0.114 ± 0.11
Phytol	25.20	2.11 ± 2.101

**TABLE 3:** Essential oil composition of *Salvia syriaca* species.

Component	Retention time (min)	%
α‐Pinene	4.79	5.55 ± 0.027
Camphene	5.08	0.91 ± 0.006
Sabinene	5.56	5.04 ± 0.001
β‐Pinene	5.63	3.25 ± 0.068
β‐Myrcene	5.89	0.161 ± 0.16
dl‐Limonene	6.69	0.96 ± 0.013
trans‐β‐Ocimene	6.87	0.191 ± 0.19
α‐Terpinolene	7.95	2.59 ± 0.018
Borneol	9.62	0.67 ± 0.026
Bornyl acetate	12.02	2.29 ± 0.074
Bicycloelemene	12.83	0.65 ± 0.115
1,5,5‐Trimethyl‐6‐methylene‐cyclohexene	13.02	4.51 ± 0.013
α‐Copaene	13.78	2.12 ± 0.012
β‐Bourbonene	13.96	0.46 ± 0.002
β‐Elemene	14.08	2.04 ± 0.011
α‐Gurjunene	14.42	0.84 ± 0.003
trans‐Caryophyllene	14.62	4.27 ± 0.0
γ‐Elemene	14.83	2.32 ± 0.021
β‐Selinene	15.23	0.62 ± 0.004
Germacrene D	15.75	16.17 ± 0.183
Tetra decamethyl cycloheptasiloxane	15.91	0.84 ± 0.004
Bicyclogermacrene	16.02	10.58 ± 0.072
α‐Farnesene	16.13	0.68 ± 0.004
6‐epi‐Shyobunol	16.43	5.07 ± 0.036
Bicyclo [4.1.0] heptan‐3‐ol, 4,7,7‐trimethyl‐, (1.alpha., 3.beta., 4.alpha., 6.alpha.)	16.52	3 ± 0.022
Germacrene B	17.06	0.79 ± 0.005
(+)‐Spathulenol	17.45	8.47 ± 0.058
Caryophyllene oxide	17.53	3.4 ± 0.023
Ledol	17.87	0.41 ± 0.01
Isospathulenol	18.44	1.53 ± 0.011
α‐Cadinol	18.70	2.07 ± 0.016
α‐Costol	18.98	0.41 ± 0.003
Shyobunol	19.31	5.52 ± 0.044
(E)‐5‐Octadecene	24.80	1.43 ± 0.016

**TABLE 4:** Essential oil composition of *Salvia palaestina* species.

Component	Retention time (min)	%
Sabinene	5.55	0.033 ± 0.032
1‐Octen‐3‐ol	5.68	0.72 ± 0.026
Isoamyl isobutanoate	6.31	0.33 ± 0.016
α‐Terpinene	6.52	0.071 ± 0.07
Cymol	6.60	0.42 ± 0.013
1,8‐Cineole	6.73	0.22 ± 0.006
trans‐β‐Ocimene	6.86	0.12 ± 0.0
cis‐β‐Ocimene	7.08	1.02 ± 0.034
γ‐Terpinene	7.31	0.25 ± 0.008
trans‐Sabinene hydrate	7.51	0.5 ± 0.016
trans‐Linalool oxide	7.61	0.13 ± 0.005
cis‐Linalool oxide	7.95	0.12 ± 0.005
Linalool	8.25	10.14 ± 0.34
Terpineol	9.85	0.9 ± 0.023
L‐α‐Terpineol	10.16	0.27 ± 0.008
Bicycloelemene	12.83	0.14 ± 0.02
1,5,5‐Trimethyl‐6‐methylene‐cyclohexene	13.02	1.2 ± 0.038
α‐Cubebene	13.26	0.14 ± 0.003
Copaene	13.80	4.09 ± 0.114
β‐Bourbonene	13.97	1.12 ± 0.027
β‐Elemene	14.09	4.22 ± 0.113
4‐(p‐Tolyl)pentanal	14.35	0.081 ± 0.08
trans‐Caryophyllene	14.68	11.97 ± 0.364
α‐Caryophyllene	15.25	1.69 ± 0.023
Germacrene D	15.41	21.45 ± 0.55
Bicyclogermacrene	16.03	3.79 ± 0.052
trans‐Cubebol	16.35	0.43 ± 0.018
δ‐Cadinene	16.45	1.87 ± 0.014
β‐Calacorene	16.81	0.071 ± 0.07
1,5‐epoxysalvial‐4 (14)‐ene	17.24	0.82 ± 0.053
(+)‐Spathulenol	17.51	2.761 ± 2.76
Caryophyllene oxide	17.58	12.53 ± 2.56
Salvial‐4 (14)‐en‐1‐one	17.71	0.52 ± 0.013
Neopentyl‐beta‐phenylpropionate	17.80	0.111 ± 0.11
(E)‐Caryophyllene	17.87	0.171 ± 0.17
3‐Cyclohexen‐1‐carboxaldehyde, 3,4‐dimethyl—	17.96	0.521 ± 0.52
α‐Humulene epoxide II	17.97	0.541 ± 0.54
γ‐Gurjunene	18.13	0.51 ± 0.033
α‐Longipinene	18.25	0.24 ± 0.023
Cadina‐1 (6), 4‐diene	18.46	1.29 ± 0.02
Isogermacrene D	18.55	0.56 ± 0.01
α‐Cadinol	18.70	2.59 ± 0.035
8‐Isopropyl‐1,5‐dimethyltricyclo [4.4.0.02, 7] dec‐4‐en‐3‐one	19.08	0.75 ± 0.038
(3S,4R,5S,6R,7S)‐Aristol‐9‐en‐3‐ol	19.22	0.99 ± 0.106
Elemol	19.55	0.4 ± 0.072
5‐(2′,2′)‐Dimethyl‐6′‐methylidenecyclohexyl‐2‐pentanone	20.10	0.21 ± 0.2
(+)‐β‐Costol	19.74	0.45 ± 0.13
Alloaromadendrene oxide‐(2)	20.47	0.45 ± 0.105
α‐Muurolene‐14‐ol	20.62	0.11 ± 0.105
14‐OH‐δ‐Cadinene	20.98	0.39 ± 0.065
Octadeamethyl‐cyclononasiloxane	21.18	0.091 ± 0.09
Hexahydrofarnesyl acetone	21.47	0.31 ± 0.088
(−)‐Neoclovene‐(I), dihydro—	21.55	0.11 ± 0.101
Phthalic acid	21.87	0.2 ± 0.001
Sclareoloxide	22.20	0.45 ± 0.007
Farnesol (E), methyl ether	22.55	0.41 ± 0.018
Nerolidol	22.74	0.24 ± 0.038
Gerany‐p‐cymene	23.10	1.17 ± 0.035
3, 4‐Dihydrobenzo[b]fluoranthene	24.59	0.041 ± 0.04
1‐Octadecene	24.80	0.22 ± 0.001
Phytol	25.20	1.86 ± 0.01
trans‐Farnesol	26.23	0.11 ± 0.047
Valeranone	26.33	0.05 ± 0.054

**TABLE 5:** Essential oil composition of *Salvia multicaulis* species.

Component	Retention time (min)	%
2‐Hexenal	3.47	0.18 ± 0.0
Tricyclo [2.2.1.0 (2,6)]heptane, 1,7,7‐trimethyl—	4.59	0.2 ± 0.004
α‐Phellandrene	4.68	0.49 ± 0.01
α‐Pinene	4.86	8.67 ± 0.07
Camphene	5.12	3.72 ± 0.03
2,4‐Thujadiene	5.20	0.09 ± 0.0
Sabinene	5.58	0.18 ± 0.01
β‐Pinene	5.66	2.85 ± 0.015
β‐Myrcene	5.90	1.46 ± 0.01
α‐Terpinene	6.45	0.21 ± 0.0
β‐Cymene	6.65	0.85 ± 0.01
1,8‐Cineole	6.80	10.48 ± 0.07
γ‐Terpinene	7.33	0.28 ± 0.0
Terpineol	7.54	0.37 ± 0.0
α‐Terpinolene	7.97	0.14 ± 0.004
trans‐Sabinene hydrate	8.22	0.93 ± 0.004
β‐Thujone	8.58	0.031 ± 0.03
2‐p‐Menthen‐1‐ol	8.73	0.25 ± 0.005
Camphor	9.25	9.18 ± 0.049
Borneol	9.47	0.12 ± 0.02
Pinocarvone	9.57	0.08 ± 0.01
Isoborneol	9.79	11.86 ± 0.02
Terpinen‐4‐ol	9.93	1.63 ± 0.034
α‐Terpineol	10.19	0.5 ± 0.069
Myrtenol	10.30	1.06 ± 0.075
Bornyl acetate	12.14	10.96 ± 0.01
Sabinyl acetate	12.20	2.51 ± 0.054
Myrtenyl acetate	12.82	0.23 ± 0.035
α‐Cubebene	13.29	0.031 ± 0.03
α‐Copaene	13.80	0.38 ± 0.045
Geranyl propionate	13.90	0.09 ± 0.005
β‐Bourbonene	13.99	0.13 ± 0.015
α‐Gurjunene	14.45	0.051 ± 0.05
trans‐Caryophyllene	14.72	9.74 ± 0.08
Germacrene D	14.82	0.26 ± 0.0
Aromandendrene	15.00	0.33 ± 0.055
α‐Humulene	15.27	0.98 ± 0.01
9‐epi‐β‐Caryophyllene	15.40	0.16 ± 0.0
γ‐Muurolene	15.65	0.16 ± 0.0
β‐Selinene	15.86	0.15 ± 0.0
Hinesene	16.00	0.59 ± 0.005
Bornyl isovalerate	16.19	0.36 ± 0.005
γ‐Cadinene	16.33	0.57 ± 0.005
δ‐Cadinene	16.47	0.99 ± 0.005
α‐Farnesene	16.54	0.27 ± 0.005
Ledane	16.77	0.66 ± 0.0
1 (7), 3, 8‐o‐Menthatriene	17.02	0.36 ± 0.005
Caryophyllene oxide	17.59	5.53 ± 0.035
γ‐Gurjunene	17.72	0.19 ± 0.005
Santalol	17.81	0.041 ± 0.04
Isoaromadendrene epoxide	17.92	0.19 ± 0.0
Humulene oxide II	17.99	0.5 ± 0.0
Alloaromadendrene oxide‐(1)	18.27	0.37 ± 0.0
Caryophylla‐4 (12)0.8 (13)‐diene‐5β‐ol	18.47	4.14 ± 0.024
β‐Eudesmol	18.69	1.71 ± 0.01
Caryophyllenol‐II	19.00	0.68 ± 0.004
ent‐Germacra‐4 (15), 5, 10 (14)‐trien‐1β‐ol	19.25	0.09 ± 0.0
Shyobunol	19.33	0.41 ± 0.005
3‐s‐Butyl‐1‐cyclohexene	21.12	0.1 ± 0.005
Phytol	25.21	0.1 ± 0.0

### 3.3 Fatty Acids of Seed Oil

Palmitic, stearic, linoleic, and linolenic acids were detected in the seed oils of all studied *Salvia* species, showing considerable quantitative variation. The highest content of palmitic acid (10.92% ± 0.33% v/w) was found in *S. multicaulis* , while *S. palaestina* exhibited the greatest amount of stearic acid (2.92% ± 0.045% v/w). *S. multicaulis* also contained the highest proportion of linoleic acid (52.57% ± 4.57% v/w), whereas *S. nemorosa* showed the maximum level of linolenic acid (46.3% ± 0.594% v/w). 8‐Octadecenoic acid and trans‐oleic acid were present in all species except *S. spinosa* . The highest concentration of 8‐octadecenoic acid (10.245% ± 10.24% v/w) was detected in *S. multicaulis* , while *S. palaestina* contained the highest level of trans‐oleic acid (13.765% ± 13.76% v/w). Cis‐oleic acid was detected only in *S. spinosa* (28.02% ± 1.075% v/w) and *S. palaestina* (11.245% ± 11.24% v/w), whereas cis‐13‐octadecenoic acid was identified only in *S. nemorosa* (0.445% ± 0.4% v/w). Eicosenoic acid was found in the seed oils of *S. nemorosa* , *S. spinosa* and *S. palaestina* . Linolenic and linoleic acids were the predominant fatty acids in *S. nemorosa* , *S. syriaca* and *S. palaestina* . In *S. spinosa* , linolenic and cis‐oleic acids represented the main fatty acids, while *S. multicaulis* was characterized by high levels of linoleic acid followed by trans‐oleic acid (Table 6).

**TABLE 6:** Fatty acids in seed oil of the studied *Salvia* species.

Fatty acid	Retention time (min)	Species				
		*S. nemorosa*	*S. spinosa*	*S. syriaca*	*S. palaestina*	*S. multicaulis*
Palmitic acid	16.651	4.7 ± 0.115	8.97 ± 0.025	7.86 ± 0.085	8.59 ± 0.23	10.92 ± 0.33
Stearic acid	22.397	2.5 ± 0.005	2.56 ± 0.004	1.95 ± 0.054	2.92 ± 0.045	1.11 ± 1.1
8‐Octadecenoic acid	24.468	9.311 ± 9.31	—	6.855 ± 6.85	0.765 ± 0.76	10.245 ± 10.24
trans‐Oleic acid	24.491	8.695 ± 8.69	—	9.04 ± 7.125	13.765 ± 13.76	12.72 ± 11.045
cis‐Oleic acid	24.497	—	28.02 ± 1.075	—	11.245 ± 11.24	—
cis‐13‐Octadecenoic acid	24.697	0.445 ± 0.4	—	—	—	—
Linoleic acid	27.738	27.1 ± 0.5	20.31 ± 0.005	36.46 ± 0.465	20.74 ± 1.075	52.57 ± 4.57
Linolenic acid	31.832	46.3 ± 0.594	39.85 ± 1.33	37.83 ± 0.05	41.34 ± 0.495	12.41 ± 4.585
Eicosenic acid	32.138	0.6 ± 0.03	0.275 ± 0.27	—	0.62 ± 0	—

## 4 Discussion

The comparative analysis of the investigated *Salvia* species revealed considerable diversity in essential oil content, seed oil yield, essential oil composition, and fatty acid profiles, highlighting the pronounced metabolic richness and chemotypic variability within the genus *Salvia*, which may be attributed to genetic factors and evolutionary adaptations. Among the examined species, *S. multicaulis* exhibited the highest essential oil yield (0.37% v/w), indicating its potential as a valuable source for essential oil extraction. In contrast, *S. nemorosa* , *S. spinosa* , and *S. palaestina* yielded low essential oil contents, suggesting their limited suitability for large‐scale essential oil extraction unless specific target compounds are desired. Previous studies have reported essential oil contents ranging from 0.17% to 0.91% in *S. multicaulis* (Morteza‐Semnani et al. 2005; Saadatjou et al. 2015; Tavakoli et al. 2022), 0.05%–0.48% in *S. nemorosa* (Chalchat et al. 2004; Chizzola 2012; Rajabi et al. 2014), 0.24%–1.35% in *S. palaestina* (Salehi et al. 2005; Gürsoy et al. 2012; Sabbobeh et al. 2015), 0.02%–0.4% in *S. spinosa* (Baher Nik and Mirza 2005; Flamini et al. 2007; Bahadori et al. 2015), and 0.035%–1.4% in *S. syriaca* (Saadatjou et al. 2015; Iravani et al. 2020; Bagheri and Yadegari 2021). On the other hand, *S. nemorosa* demonstrated the highest seed oil content (53.3% v/w), positioning it as a promising candidate for edible or industrial oil production. Previous studies have reported that the seed oil content ranges from 33.86% to 39.54% in *S. multicaulis* (Nejad Habibvash et al. 2007; Moazzami Farida et al. 2016), 14%–48.7% in *S. nemorosa* (Nejad Habibvash et al. 2007; Moazzami Farida et al. 2016; Sepehry Javan et al. 2019), 23.17%–34.03% in *S. spinosa* (Moazzami Farida et al. 2016), and 22.7%–32.34% in *S. syriaca* (Bagci et al. 2004; Nejad Habibvash et al. 2007; Moazzami Farida et al. 2016). However, no data were found regarding the seed oil content of *S. palaestina* . Interestingly, no consistent correlation was observed between essential oil and seed oil contents, suggesting independent regulation of these traits possibly via distinct biosynthetic pathways, which may be exploited for dual‐purpose breeding. The number of identified compounds in the essential oils varied markedly among the *Salvia* species, ranging from 23 in *S. nemorosa* to 68 in *S. spinosa* . Each species exhibited a distinct chemical profile, characterized by unique dominant constituents. *S. multicaulis* was particularly rich in monoterpenes such as isoborneol and bornyl acetate, compounds commonly associated with bioactive properties including respiratory benefits, anti‐inflammatory effects, and aromatic applications (Yang et al. 2014). Previous investigations have reported diverse dominant constituents in the essential oil of *S. multicaulis* . For instance, Senatore et al. (2004) identified α‐copaene, α‐pinene, myrtenol, and trans‐sabinyl acetate as major components. Morteza‐Semnani et al. (2005) found camphor, 1,8‐cineole, borneol, and α‐pinene as dominant compounds, while Amiri (2012) reported borneol, bornyl acetate, camphor, β‐caryophyllene, and camphene as the most abundant constituents. According to Ramezani et al. (2015), camphene, α‐pinene, camphor, limonene, 1,8‐cineole, β‐pinene, and bornyl acetate were the major components. Bagci et al. (2019) identified α‐pinene, α‐eudesmol, valeranone, and camphene as dominant components, whereas Najafian et al. (2020) reported α‐pinene, 1,8‐cineole, camphene, β‐pinene, camphor, borneol, bornyl acetate, and (E)‐caryophyllene as the major compounds. Tavakoli et al. (2022) identified α‐pinene, α‐phellandrene, 1,8‐cineole, bornyl acetate, and β‐caryophyllene as the principal constituents. Both *S. syriaca* and *S. palaestina* exhibited high levels of germacrene D, a sesquiterpene recognized for its antimicrobial and antioxidant activities, highlighting their potential for pharmaceutical and cosmetic applications (Victoria et al. 2012; Sitarek et al. 2017). Consistent with our findings, several previous studies have also reported germacrene D as one of the main components of *S. syriaca* essential oil (Sefidkon and Mirza 1999; Karamian et al. 2014; Iravani et al. 2020). However, in other investigations, germacrene D was not detected in this species; instead, compounds such as 1,8‐cineole and camphor (Forouzin et al. 2015), as well as cis‐thujone, camphor, trans‐thujone, and 1,8‐cineole (Bagheri and Yadegari 2021), have been reported as the predominant constituents. Similar to *S. syriaca* , germacrene D has also been reported as one of the dominant components in the essential oil of *S. palaestina* (Salehi et al. 2005; Rustaiyan et al. 2005; Al‐Jaber et al. 2012; Fattahi et al. 2014). However, some studies did not detect germacrene D in this species (Gürsoy et al. 2012; Sabbobeh et al. 2015). *S. spinosa* exhibited a distinct chemical profile dominated by esters such as isopentyl isovalerate and 2‐methylbutyl isovalerate, suggesting a divergent metabolic pathway that could be exploited for targeted industrial uses. Isopentyl isovalerate has also been identified as a major component of *S. spinosa* essential oil in other studies (Baher Nik and Mirza 2005; Flamini et al. 2007). Nevertheless, contrasting results have been reported in other research: Bahadori et al. (2015) identified caryophyllene oxide, spathulenol, linalool, and trans‐caryophyllene as the main constituents, while Hadipanah et al. (2015) found α‐terpinolene, β‐ocimene, β‐patchoulene, β‐ bourbonene, and 1,8‐cineole as the dominant compounds in this species. In *S. nemorosa* , sesquiterpenes such as (+)‐spathulenol, trans‐caryophyllene, and caryophyllene oxide were identified as the major components, indicating potential therapeutic properties including anti‐inflammatory and antimicrobial effects (Dahham et al. 2015; do Nascimento et al. 2018). Previous studies have reported various dominant constituents in the essential oil of *S. nemorosa* , including caryophyllene oxide, caryophyllene, germacrene D, and sabinene (Chalchat et al. 2004); caryophyllene oxide, spathulenol, and β‐phellandrene (Malenčić et al. 2004); sabinene, caryophyllene, and germacrene D (Coisin et al. 2012); sabinene, germacrene D, caryophyllene, and caryophyllene oxide (Chizzola 2012); caryophyllene, spathulenol, caryophyllene oxide, linalool, and germacrene D (Rajabi et al. 2014); as well as spathulenol, caryophyllene oxide, and caryophyllene (Mahdieh et al. 2018). These findings are largely consistent with our results.

The fatty acid analysis revealed that all studied *Salvia* species contained palmitic, stearic, linoleic, and linolenic acids, although in varying proportions. *S. multicaulis* stood out for its high levels of linoleic and palmitic acids, indicating suitability for food and cosmetic industry applications due to functional and oxidative stability properties. Consistent with these findings, Keser et al. (2015) also reported oleic acid (29.23%) and palmitic acid (20.47%) as the predominant fatty acids in the seed oil of *S. multicaulis* . *S. nemorosa* exhibited notably high concentrations of linolenic acid, an omega‐3 fatty acid recognized for cardiovascular benefits, underscoring its potential as a nutritionally valuable oil source (Fleming and Kris‐Etherton 2014). Similarly, Moazzami Farida et al. (2016) identified linolenic acid (49.28%) as the major fatty acid in the seed oil of *S. nemorosa* , whereas Sepehry Javan et al. (2019) reported erucic acid (26.9%) as the dominant fatty acid in this species. In agreement with the present findings, Bagci et al. (2004) and Gören et al. (2006), identified linoleic and linolenic acids as the predominant fatty acids in the seed oil of *S. syriaca* . The detection of unique fatty acids in particular species, such as cis‐oleic acid detected only in *S. spinosa* and *S. palaestina* , and cis‐13‐octadecenoic acid identified exclusively in *S. nemorosa* , highlights a species‐specific lipid profile, which could guide targeted exploitation in nutraceutical, cosmetic, or bio‐lubricant applications. The high levels of trans‐oleic acid in *S. palaestina* and 8‐octadecenoic acid in *S. multicaulis* may contribute to the enhanced stability and potential functional applications for their oils in food and cosmetic formulations (Parker et al. 2024; Kato et al. 2025). Additionally, the presence of eicosenoic acid in *S. nemorosa* , *S. spinosa* , and *S. palaestina* suggests their potential use in the production of bio‐lubricants and surface‐active agents. Differences observed between our findings and previous reports may be explained by variations in genotype, environmental conditions, harvest time, extraction methods, and analytical techniques. The observed phytochemical diversity among the studied *Salvia* species highlights their wide‐ranging potential applications, from medicinal uses to the fragrance and cosmetic industries.

## 5 Conclusions

This study revealed notable diversity among the studied *Salvia* species in essential oil yield, seed oil content, and chemical composition. *S. multicaulis* exhibited the highest essential oil content, rich in monoterpenes such as isoborneol and bornyl acetate, highlighting its suitability for pharmaceutical and aromatic applications. In contrast, *S. nemorosa* showed the highest seed oil content, with a high linolenic acid level, indicating its potential for nutritional and industrial uses. The variation in dominant essential oil constituents from germacrene D in *S. syriaca* and *S. palaestina* to caryophyllene derivatives in *S. nemorosa* reflects significant chemotypic diversity within the genus. These findings highlight the potential of *Salvia* species as valuable sources of bioactive compounds and provide a foundation for domestication and breeding programs.

## Author Contributions

**Yasaman Veis Mohammadi:** investigation (lead). **Jalal Khorshidi:** methodology (lead), project administration (lead), software (lead), writing – original draft (lead). **Farzad Nazari:** supervision (lead).

## Conflicts of Interest

The authors declare no conflicts of interest.

## Data Availability

All data are incorporated into the article.

## References

[fsn371308-bib-0001] Abou Baker, D. H. , R. Amarowicz , A. Kandeil , M. Ali , and E. Ibrahim . 2021. “Antiviral Activity of *Lavandula angustifolia* L. and *Salvia officinalis* L. Essential Oils Against Avian Influenza H5N1 Virus.” Journal of Agriculture and Food Research 4: 100135. 10.1016/j.jafr.2021.100135. 36570026 PMC9767472

[fsn371308-bib-0002] Al‐Jaber, H. I. , M. A. Al‐Qudah , L. M. Barhoumi , I. F. Abaza , and F. U. Afifi . 2012. “Essential Oil Composition of the Aerial Parts of Fresh and Air‐Dried *Salvia palaestina* Benth. (Lamiaceae) Growing Wild in Jordan.” Natural Product Research 26, no. 13: 1179–1187. 10.1080/14786419.2010.543901. 21861642

[fsn371308-bib-0003] Amiri, H. 2012. “Chemical Composition and Antioxidant Activity of the Essential Oil and Methanolic Extract of *Salvia Multicaulis* Vahl.” Journal of Medicinal Plants 11, no. 41: 111–117.

[fsn371308-bib-0004] Askari, S. F. , R. Avan , Z. Tayarani‐Najaran , A. Sahebkar , and S. Eghbali . 2021. “Iranian *Salvia* Species: A Phytochemical and Pharmacological Update.” Phytochemistry 183: 112619. 10.1016/j.phytochem.2020.112619. 33373790

[fsn371308-bib-0005] Bagci, E. , E. Akbaba , C. Maniu , E. Ungureanu , and L. Hritcu . 2019. “Evaluation of Antiamnesic Activity of *Salvia Multicaulis* Essential Oil on Scopolamine‐Induced Amnesia in Rats: In Vivo and In Silico Approaches.” Heliyon 5: e02223. 10.1016/j.heliyon.2019.e02223. 31440590 PMC6698886

[fsn371308-bib-0006] Bagci, E. , M. Vural , T. Dirmenci , L. Bruehl , and K. Aitzetmüller . 2004. “Fatty Acid and Tocochromanol Patterns of Some *Salvia* L. Species.” Zeitschrift Für Naturforschung. C, A Journal of Biosciences 59, no. 5–6: 305–309. 10.1515/znc-2004-5-601. 18998390

[fsn371308-bib-0007] Bagheri, F. , and M. Yadegari . 2021. “Properties of Essential Oil of *Salvia syriaca* L. Under Different Phenological Stages and Climatic Conditions in Chaharmahal and Bakhtiari Province.” Journal of Plant Environmental Physiology 15: 80–93. 10.30495/iper.2021.679517.

[fsn371308-bib-0008] Bahadori, M. B. , H. Valizadeh , B. Asghari , L. Dinparast , M. Moridi Farimani , and S. Bahadori . 2015. “Chemical Composition and Antimicrobial, Cytotoxicity, Antioxidant and Enzyme Inhibitory Activities of *Salvia spinosa* L.” Journal of Functional Foods 18: 727–736. 10.1016/j.jff.2015.09.011.

[fsn371308-bib-0009] Baher Nik, Z. , and M. Mirza . 2005. “Volatile Constituents of *Salvia spinosa* L. From Iran.” Flavour and Fragrance Journal 20, no. 3: 311–312. 10.1002/ffj.1419.

[fsn371308-bib-0010] Cegiełka, A. , M. Chmiel , E. Hać‐Szymańczuk , and D. Pietrzak . 2022. “Evaluation of the Effect of Sage (*Salvia officinalis* L.) Preparations on Selected Quality Characteristics of Vacuum‐Packed Chicken Meatballs Containing Mechanically Separated Meat.” Applied Sciences 12, no. 24: 12890. 10.3390/app122412890.

[fsn371308-bib-0011] Chalchat, J. C. , D. P. Silvana , A. M. Zoran , and S. G. Momcilo . 2004. “Composition of Essential Oils of Some Wild *Salvia* Species Growing in Serbia.” Journal of Essential Oil Research 16, no. 5: 476–478. 10.1080/10412905.2004.9698775.

[fsn371308-bib-0012] Chizzola, R. 2012. “Composition and Variability of the Essential Oil of *Salvia nemorosa* (Lamiaceae) From the Vienna Area of Austria.” Natural Product Communications 7, no. 12: 1671–1672. 10.1177/1934578X1200701232. 23413579

[fsn371308-bib-0013] Coisin, M. , I. Burzo , M. Stefan , E. Rosenhech , and M. M. Zamfirache . 2012. “Chemical Composition and Antibacterial Activity of Essential Oils of Three *Salvia* Species, Widespread in Eastern Romania. *Analele Ştiinţifice Ale Universităţii Al. I. Cuza* .” Iaşi s. II a. Biologie Vegetală 58, no. 1: 51–58.

[fsn371308-bib-0014] Dahham, S. S. , Y. M. Tabana , M. A. Iqbal , et al. 2015. “The Anticancer, Antioxidant and Antimicrobial Properties of the Sesquiterpene β‐Caryophyllene From the Essential Oil of *Aquilaria crassna* .” Molecules 20, no. 7: 351–358. 10.3390/molecules200711808. PMC6331975 26132906

[fsn371308-bib-0015] Demirpolat, A. 2023. “Essential Oil Composition Analysis, Antimicrobial Activities, and Biosystematic Studies on Six Species of *Salvia* .” Life 13: 634. 10.3390/life13030634. 36983789 PMC10054517

[fsn371308-bib-0016] do Nascimento, K. F. , F. M. F. Moreira , J. Alencar Santos , et al. 2018. “Antioxidant, Anti‐Inflammatory, Antiproliferative and Antimycobacterial Activities of the Essential Oil of *Psidium guineense* Sw. and Spathulenol.” Journal of Ethnopharmacology 210: 351–358. 10.1016/j.jep.2017.08.030. 28844678

[fsn371308-bib-0017] Eidi, A. , and M. Eidi . 2009. “Antidiabetic Effects of Sage (*Salvia officinalis* L.) Leaves in Normal and Streptozotocin‐Induced Diabetic Rats.” Diabetes and Metabolic Syndrome: Clinical Research and Reviews 3: 40–44. 10.1016/j.dsx.2008.10.007.

[fsn371308-bib-0018] Fattahi, B. , V. Nazeri , S. Kalantari , and M. Bonfill . 2014. “Identification of Compounds in the Essential Oil and Quantification of Flavonoids and Rosmarinic Acid in *Salvia reuterana* Boiss. and *Salvia palaestina* Benth.” Iranian Journal of Medicinal and Aromatic Plants Research 30, no. 3: 463–475. 10.22092/ijmapr.2014.7682.

[fsn371308-bib-0019] Flamini, G. , P. L. Cioni , I. Morelli , and A. Bader . 2007. “Essential Oils of the Aerial Parts of Three *Salvia* Species From Jordan: *Salvia lanigera* , *S. Spinosa* and *S. syriaca* .” Food Chemistry 100, no. 2: 732–735. 10.1016/j.foodchem.2005.10.032.

[fsn371308-bib-0020] Fleming, J. A. , and P. M. Kris‐Etherton . 2014. “The Evidence for α‐Linolenic Acid and Cardiovascular Disease Benefits: Comparisons With Eicosapentaenoic Acid and Docosahexaenoic Acid.” Advances in Nutrition 5, no. 6: 863S–876S. 10.3945/an.114.005850. 25398754 PMC4224228

[fsn371308-bib-0021] Forouzin, F. , R. Jamei , and R. Heidari . 2015. “Comparison of Essential Oil Components and Antioxidant Activity Between *Salvia Syriaca* and *Salvia Aristata* in Their Natural Habitats in West Azerbaijan Province, Iran.” Journal of Pharmacy and Pharmacology 3, no. 8: 400–404. 10.17265/2328-2150/2015.08.006.

[fsn371308-bib-0022] Ghorbani, A. , and M. Esmaeilizadeh . 2017. “Pharmacological Properties of *Salvia Officinalis* and Its Components.” Journal of Traditional and Complementary Medicine 7, no. 4: 433–440. 10.1016/j.jtcme.2016.12.014. 29034191 PMC5634728

[fsn371308-bib-0023] Gören, A. C. , T. Kiliç , T. Dirmenci , and G. Bilsel . 2006. “Chemotaxonomic Evaluation of Turkish Species of *Salvia*: Fatty Acid Compositions of Seed Oils.” Biochemical Systematics and Ecology 34, no. 2: 160–164. 10.1016/j.bse.2005.09.002.

[fsn371308-bib-0024] Gürsoy, N. , B. Tepe , and H. A. Akpulat . 2012. “Chemical Composition and Antioxidant Activity of the Essential Oils of *Salvia palaestina* (Bentham) and *S. Ceratophylla* (L.).” Records of Natural Products 6: 278–287.

[fsn371308-bib-0025] Guy, P. , P. Kamatou , A. M. Viljoen , and P. Steenkamp . 2010. “Antioxidant, Antiinflammatory Activities and HPLC Analysis of South African *Salvia* Species.” Food Chemistry 119: 684–688. 10.1016/j.foodchem.2009.07.010.

[fsn371308-bib-0026] Hadipanah, A. , A. R. Golparvar , A. M. Mehrabi , and M. Jafarpour . 2015. “Identification of the Volatile Composition of *Stachys Lavandulifolia* Vahl. and *Salvia spinosa* L. in Isfahan Climatic Conditions.” Journal of Biodiversity and Environmental Sciences 6, no. 6: 187–193.

[fsn371308-bib-0027] Hatipoglu, S. D. , N. Zorlu , T. Dirmenci , A. C. Goren , T. Ozturk , and G. Topcu . 2016. “Determination of Volatile Organic Compounds in Fourty Five *Salvia* Species by Thermal Desorption‐GC‐MS Technique.” Records of Natural Products 10: 659–700.

[fsn371308-bib-0028] Iravani, M. , R. Mahinpour , Z. Zahraei , and Z. Toluei . 2020. “Study on the Quantitative and Qualitative Diversity of Essential Oil in Four Wild *Salvia* spp. in Kashan Region.” Iranian Journal of Medicinal and Aromatic Plants Research 36: 642–654. 10.22092/ijmapr.2020.342118.2735.

[fsn371308-bib-0029] Jazayeri Gharehbagh, H. , M. Ebrahimi , F. Dabaghian , et al. 2023. “Chemical Composition, Cholinesterase, and α‐Glucosidase Inhibitory Activity of the Essential Oils of Some Iranian Native *Salvia* Species.” BMC Complementary Medicine and Therapies 23: 184. 10.1186/s12906-023-04004-w. 37270541 PMC10239571

[fsn371308-bib-0030] Karamian, R. , M. Asadbegy , and R. Pakazad . 2014. “Essential Oil Compositions, Antioxidant and Antibacterial Activities of Two *Salvia* Species (*S. Grossheimii* Bioss. and *S. syriaca* L.) Growing in Iran.” Journal of Essential Oil Bearing Plants 17, no. 2: 331–345. 10.1080/0972060X.2014.895156.

[fsn371308-bib-0031] Kato, A. , E. Shimizu , H. Tsujimura , et al. 2025. “A Possible Role of Cis‐8‐Octadecenoic Acid of the Sebum in Facial Skin Redness.” Journal of Cosmetic Dermatology 24: e16570. 10.1111/jocd.16570. 39279230 PMC11743226

[fsn371308-bib-0032] Keser, S. , S. Celik , S. Turkoglu , Ö. Yilmaz , and I. Turkoglu . 2015. “Vitamin, Sterol and Fatty Acid Contents of Some Edible and Medicinal Plants From East and Southeast Anatolia (Turkey).” Turkish Journal of Pharmaceutical Sciences 12, no. 2: 46–59. 10.5505/tjps.2015.66375.

[fsn371308-bib-0033] Khorshidi, J. , S. H. Amanollahi , and P. Bayazidi Azar . 2024. “Optimization of Essential Oil Extraction From *Satureja Sahendica* Bornm.” Journal of Food Science and Technology (Iran) 21, no. 148: 103–111. 10.22034/FSCT.21.148.103.

[fsn371308-bib-0034] Khorshidi, J. , M. R. Morshedloo , and S. Moradi . 2020. “Essential Oil Composition of Three Iranian *Hypericum* Species Collected From Different Habitat Conditions.” Biocatalysis and Agricultural Biotechnology 28: 101755. 10.1016/j.bcab.2020.101755.

[fsn371308-bib-0035] Kurşat, M. , A. Sari , Ş. Civelek , İ. Emre , and Ö. Yilmaz . 2013. “Seed Fatty Acid Amounts of Some *Salvia* L. Taxa in Elazig.” Turkish Journal of Science & Technology 8, no. 2: 115–119.

[fsn371308-bib-0036] Lopresti, A. L. 2017. “ *Salvia* (Sage): A Review of Its Potential Cognitive‐Enhancing and Protective Effects.” Drugs in R&D 17: 53–64. 10.1007/s40268-016-0157-5. 27888449 PMC5318325

[fsn371308-bib-0037] Mahdieh, M. , S. M. Talebi , and M. Akhani . 2018. “Infraspecific Essential Oil and Anatomical Variations of *Salvia nemorosa* L. (Labiatae) Populations in Iran.” Industrial Crops and Products 123: 35–45. 10.1016/j.indcrop.2018.06.061.

[fsn371308-bib-0038] Malenčić, D. J. , M. Couladis , N. Mimica‐Dukić , M. Popović , and P. Boža . 2004. “Essential Oils of Three *Salvia* Species From the Pannonian Part of Serbia.” Flavour and Fragrance Journal 19, no. 3: 225–228. 10.1002/ffj.1291.

[fsn371308-bib-0039] Mizi, L. , S. Cofrades , R. Bou , et al. 2019. “Antimicrobial and Antioxidant Effects of Combined High Pressure Processing and Sage in Beef Burgers During Prolonged Chilled Storage.” Innovative Food Science & Emerging Technologies 51: 32–40. 10.1016/j.ifset.2018.04.010.

[fsn371308-bib-0040] Moazzami Farida, S. H. , T. Radjabian , M. Ranjbar , S. A. Salami , N. Rahmani , and A. Ghorbani . 2016. “Fatty Acid Patterns of Seeds of Some *Salvia* Species From Iran – A Chemotaxonomic Approach.” Chemistry & Biodiversity 13: 451–458. 10.1002/cbdv.201500147. 26988735

[fsn371308-bib-0041] Morteza‐Semnani, K. , K. Moshiri , and M. Akbarzadeh . 2005. “The Essential Oil Composition of *Salvia Multicaulis* Vahl.” Journal of Essential Oil Bearing Plants 8, no. 1: 6–10. 10.1080/0972060X.2005.10643412.

[fsn371308-bib-0042] Naghibi, F. , M. Mosaddegh , M. Mohammadi Motamed , and A. Ghorbani . 2005. “Labiatae Family in Folk Medicine in Iran: From Ethnobotany to Pharmacology.” Iranian Journal of Pharmaceutical Research 2: 63–79. 10.22037/ijpr.2010.619.

[fsn371308-bib-0043] Najafian, S. , V. Rowshan , and S. Fathi . 2020. “Comparison of Chemical Constituents of Essential Oil From Aerial Constituent of *Mentha Longifolia* and *Salvia Multicaulis* Obtained by Hydro Distillation and Headspace Analysis.” Journal of Essential Oil Bearing Plants 23, no. 4: 719–727. 10.1080/0972060X.2020.1824687.

[fsn371308-bib-0044] Nejad Habibvash, F. , M. A. Rajamand , R. Heidari , S. Hosseini , and M. Heidari Ricani . 2007. “Chemical Analysis of Some *Salvia* Species Native to West Azarbaijan (Iran).” Pakistan Journal of Biological Sciences 10: 3516–3524. 10.3923/pjbs.2007. 19093457

[fsn371308-bib-0045] Ortiz‐Mendoza, N. , E. Aguirre‐Hernández , I. Fragoso‐Martínez , M. E. González‐Trujano , F. A. Basurto‐Peña , and M. J. Martínez‐Gordillo . 2022. “A Review on the Ethnopharmacology and Phytochemistry of the Neotropical Sages (*Salvia* Subgenus *Calosphace*; Lamiaceae) Emphasizing Mexican Species.” Frontiers in Pharmacology 13: 867892. 10.3389/fphar.2022.867892. 35517814 PMC9061990

[fsn371308-bib-0046] Parker, L. , K. Ward , T. Pilarski , et al. 2024. “Development and Large‐Scale Production of High‐Oleic Acid Oil by Fermentation of Microalgae.” Fermentation 10: 566. 10.3390/fermentation10110566.

[fsn371308-bib-0047] Rajabi, Z. , M. Ebrahimi , M. Farajpour , M. Mirza , and H. Ramshini . 2014. “Compositions and Yield Variation of Essential Oils Among and Within Nine *Salvia* Species From Various Areas of Iran.” Industrial Crops and Products 61: 233–239. 10.1016/j.indcrop.2014.06.038.

[fsn371308-bib-0048] Ramezani, S. , A. Abbasi , A. Shojaeiyan , N. Ahmadi , R. Cozzolino , and S. Piacente . 2015. “Extraction and Identification of Volatile Components of Two *Salvia* Species Native to Iran (*Salvia Limbata* and *S. multicaulis*) by Using Solid Phase Micro‐Extraction Method.” Journal of Horticultural Science 29, no. 3: 466–473. 10.22067/jhorts4.v0i0.40346.

[fsn371308-bib-0049] Rustaiyan, A. , M. R. Akhgar , S. Masoudi , and F. Nematollahi . 2005. “Chemical Composition of Essential Oils of Three *Salvia* Species Growing Wild in Iran: *Salvia Rhytidea* Benth., *S. limbata* C.A. Mey. and *S. palaestina* Benth.” Journal of Essential Oil Research 17, no. 5: 522–524. 10.1080/10412905.2005.9698982.

[fsn371308-bib-0050] Saadatjou, B. , A. Mohammadkhani , K. Saeidi , and H. A. Shirmardi . 2015. “The Evaluation of Morphological Variation and Essential Oil Content of *Salvia* Species Ecotypes in South‐West of Iran.” Journal of Applied Crop Breeding 3: 125–135.

[fsn371308-bib-0051] Sabbobeh, R. , H. Hejaz , H. Al‐Jaas , A. Jahajha , and S. Abu‐Lafi . 2015. “Phytochemical Analysis of Cultivated and Wild *Salvia palaestina* Using GC‐MS: A Comparative Study.” World Journal of Pharmaceutical Sciences 3, no. 12: 2348–2356.

[fsn371308-bib-0052] Salehi, P. , F. Sefidkon , L. Bazzaz Tolami , and A. Sonboli . 2005. “Essential Oil Composition of *Salvia palaestina* Benth. From Iran.” Flavour and Fragrance Journal 20, no. 5: 525–527. 10.1002/ffj.1448.

[fsn371308-bib-0053] Sefidkon, F. , and M. Mirza . 1999. “Chemical Composition of the Essential Oils of Two *Salvia* Species From Iran, *Salvia virgata* Jacq. and *Salvia syriaca* L.” Flavour and Fragrance Journal 14, no. 1: 45–46. 10.1002/(SICI)1099-1026(199901/02)14:1<45::AID-FFJ774>3.0.CO;2-S.

[fsn371308-bib-0054] Semiz, G. , D. Mutlu , B. Günal , A. Semiz , and Ş. Arslan . 2023. “The Anticancer Effect of *Salvia Pisidica* Essential Oil Through Promotion Intrinsic and Extrinsic Apoptosis Pathways in Human Cancer Cell Lines.” Journal of Herbal Medicine 39: 100664. 10.1016/j.hermed.2023.100664.

[fsn371308-bib-0055] Senatore, F. , N. A. Arnold , and F. Piozzi . 2004. “Chemical Composition of the Essential Oil of *Salvia Multicaulis* Vahl. Var. *Simplicifolia* Boiss. Growing Wild in Lebanon.” Journal of Chromatography A 1052, no. 1–2: 237–240. 10.1016/j.chroma.2004.08.095. 15527145

[fsn371308-bib-0056] Seol, G. H. , H. S. Shim , P. J. Kim , et al. 2010. “Antidepressant‐Like Effect of *Salvia sclarea* Is Explained by Modulation of Dopamine Activities in Rats.” Journal of Ethnopharmacology 130, no. 1: 187–190. 10.1016/j.jep.2010.04.035. 20441789

[fsn371308-bib-0057] Sepehry Javan, Z. , F. Rahmani , and L. Asadyar . 2019. “Fatty Acid Composition of Seed Oils of Eight *Salvia* Species Growing in Iran.” Specialty Journal of Biological Sciences 5, no. 2: 19–26.

[fsn371308-bib-0058] Shanaida, M. , N. Hudz , M. Biaton , et al. 2021. “Chromatographic Profiles and Antimicrobial Activity of the Essential Oils Obtained From Some Species and Cultivars of the Mentheae Tribe (Lamiaceae).” Saudi Journal of Biological Sciences 28, no. 11: 6145–6152. 10.1016/j.sjbs.2021.06.068. 34759738 PMC8568706

[fsn371308-bib-0059] Sitarek, P. , P. Rijo , C. Garcia , et al. 2017. “Antibacterial, Anti‐Inflammatory, Antioxidant, and Antiproliferative Properties of Essential Oils From Hairy and Normal Roots of *Leonurus sibiricus* L. and Their Chemical Composition.” Oxidative Medicine and Cellular Longevity 7384061: 7384061. 10.1155/2017/7384061. PMC5278227 28191277

[fsn371308-bib-0060] Tavakoli, M. , S. Soltani , M. Tarkesh Esfahani , and R. Karamian . 2022. “Study on Some Environmental Factors Effects on *Salvia multicaulis* Vahl. Essential Oil Composition in Hamadan Province.” Iranian Journal of Medicinal and Aromatic Plants Research 38: 545–563. 10.22092/ijmapr.2022.358508.3162.

[fsn371308-bib-0061] Victoria, F. N. , E. J. Lenardão , L. Savegnago , et al. 2012. “Essential Oil of the Leaves of *Eugenia uniflora* L.: Antioxidant and Antimicrobial Properties.” Food and Chemical Toxicology 50, no. 8: 2668–2674. 10.1016/j.fct.2012.05.002. 22583648

[fsn371308-bib-0062] Yang, H. , R. Zhao , H. Chen , P. Jia , L. Bao , and H. Tang . 2014. “Bornyl Acetate Has an Anti‐Inflammatory Effect in Human Chondrocytes via Induction of IL‐11.” IUBMB Life 66, no. 12: 854–859. 10.1002/iub.1338. 25545915

